# The Essential Role of Light-Induced Autophagy in the Inner Choroid/Outer Retinal Neurovascular Unit in Baseline Conditions and Degeneration

**DOI:** 10.3390/ijms24108979

**Published:** 2023-05-19

**Authors:** Roberto Pinelli, Michela Ferrucci, Caterina Berti, Francesca Biagioni, Elena Scaffidi, Violet Vakunseth Bumah, Carla L. Busceti, Paola Lenzi, Gloria Lazzeri, Francesco Fornai

**Affiliations:** 1 Switzerland Eye Research Institute (SERI), 6900 Lugano, Switzerland; 2 Human Anatomy, Department of Translational Research and New Technologies in Medicine and Surgery, University of Pisa, 56126 Pisa, Italy; 3 Istituto di Ricovero e Cura a Carattere Scientifico (IRCCS), 86077 Pozzili, Italy; 4 Department of Chemistry and Biochemistry College of Sciences San Diego State University, San Diego, CA 92182, USA; 5 Department of Chemistry and Physics, University of Tennessee, Knoxville, TN 37996, USA

**Keywords:** age-related macular degeneration, photo-biomodulation, choriocapillaris, retinal pigment epithelium, Bruch’s membrane, endothelium, pericytes, light exposure, nutraceuticals, photo-sensitive phytochemicals

## Abstract

The present article discusses the role of light in altering autophagy, both within the outer retina (retinal pigment epithelium, RPE, and the outer segment of photoreceptors) and the inner choroid (Bruch’s membrane, BM, endothelial cells and the pericytes of choriocapillaris, CC). Here autophagy is needed to maintain the high metabolic requirements and to provide the specific physiological activity sub-serving the process of vision. Activation or inhibition of autophagy within RPE strongly depends on light exposure and it is concomitant with activation or inhibition of the outer segment of the photoreceptors. This also recruits CC, which provides blood flow and metabolic substrates. Thus, the inner choroid and outer retina are mutually dependent and their activity is orchestrated by light exposure in order to cope with metabolic demand. This is tuned by the autophagy status, which works as a sort of pivot in the cross-talk within the inner choroid/outer retina neurovascular unit. In degenerative conditions, and mostly during age-related macular degeneration (AMD), autophagy dysfunction occurs in this area to induce cell loss and extracellular aggregates. Therefore, a detailed analysis of the autophagy status encompassing CC, RPE and interposed BM is key to understanding the fine anatomy and altered biochemistry which underlie the onset and progression of AMD.

## 1. Introduction

### 1.1. General Relevance of Light-Induced Autophagy in the Outer Retina/Inner Choroid Neurovascular Unit

**Figure 1:**
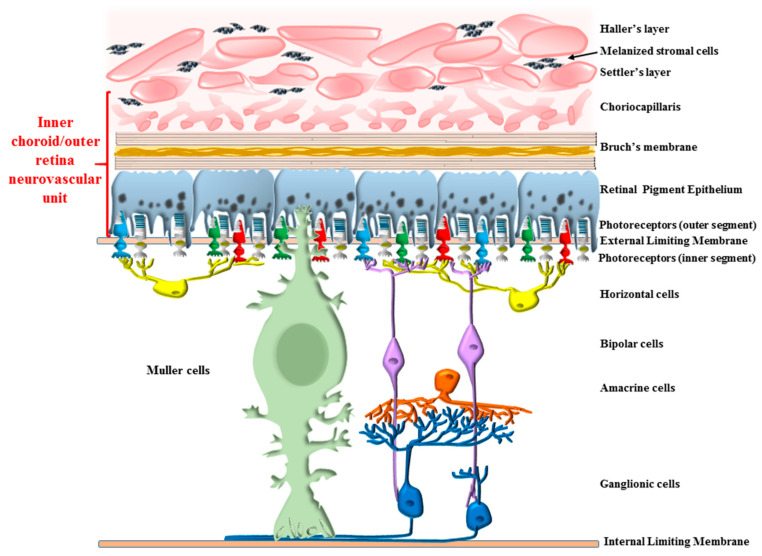
Anatomical placement of the inner choroid/outer retinal segment. The diagram focuses on structures placed at the interface between choroid and retina. These are anatomically connected and functionally bond together to cluster in a metabolic unit, the inner choroid/outer retina neurovascular unit (shown in red bracket). These structures include the choriocapillaris (CC), where pericytes and endothelial cells occur, separated from retinal pigment epithelium (RPE) by the Bruch’s membrane (BM). In this functional unit, RPE cells embrace the outer segment of photoreceptors. The metabolic demand of these connected compartments is driven by the amount of light, since this promotes photo-transduction in the photoreceptors, protein and organelle turn-over in the RPE cells, and blood supply and trophic support from the CC. All these activities, which are unique for each cell type, seem to be orchestrated synergistically by the tuning of autophagy flux. The main scope of the review is to analyze in depth the autophagy-dependent interactions in this area.

The autophagy machinery is fundamental in promoting visual processing and preserves the integrity of the outer retinal segments. This is mainly due to light-dependent biochemical cascades placed downstream of autophagy activation, which consist of various pathways mostly placed within the retinal pigment epithelium (RPE). Nonetheless, other structures placed at the choroid–retinal border are modulated by light, when regarding the autophagy machinery. In fact, autophagy is abundant at this level, where actual photo-transduction takes place, and is in need of trophic support and high protein/organelle turn-over to sustain light stimulation. This contributes to defining the inner choroid/outer retina as a sort of morpho-functional unit, which may be termed the retinal neurovascular unit (red bracket of Figure 1).

**Figure 2:**
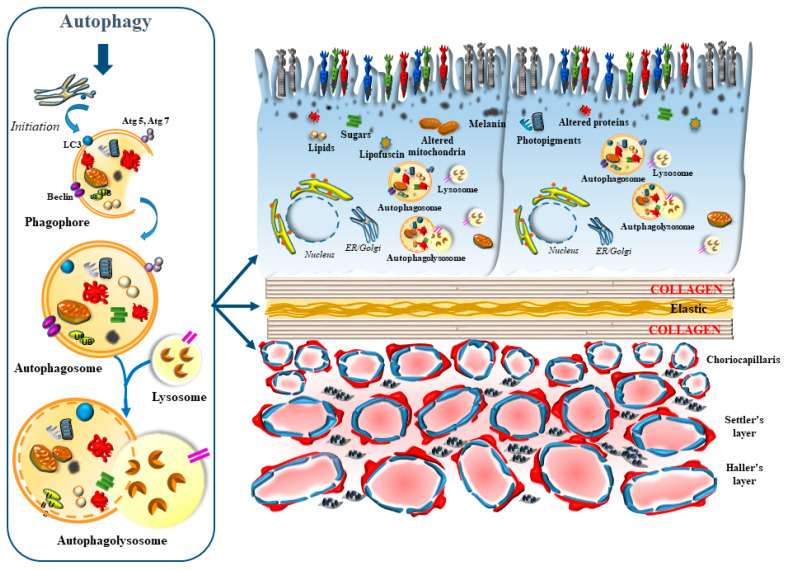
The autophagy pathway sustains the integrity of the inner choroid/outer retina segment. In the retina, the highest autophagy activity takes place in the outer regions and mostly within RPE cells [1]. These cells feature the highest amount of autophagy-related organelles and the highest autophagy turn-over. In fact, the staining of autophagy-related proteins, such as LC3, and the amount of small lysosomes, autophagosomes and the merging between these organelles to form auto-phagolysosomes, is elevated. These autophagy-related structures are responsible for clearing the cells from altered mitochondria, sugars, lipid droplets, misfolded proteins, lipofuscin, melanin, and photopigments, along with the aged outer segment of photoreceptors (the latter occurring during a process defined as LC3-associated phagocytosis, LAP, which is described in Section 2.1). Similarly, autophagy is key in the choriocapillaris, where it plays an essential role in fostering retinal integrity and the proper structure of the Bruch’s membrane.

This was analyzed in the recent elegant study by Ramachandra Rao and Fliesler [1] showing ongoing autophagy in the whole retina, including neurons, glia and endothelial cells. In this study, the amount of autophagy prevails at large in the outer retinal segment (Figure 2). This corresponds to the site, where the turn-over of autophagy-related substrates need to be stronger and faster. Such site-specificity is related to the impact of light on photoreceptors and RPE cells, which in turn strongly depends on the inner choroid, along with endothelial cells and pericytes. At this level, light-induced autophagy is mediated by reactive oxygen species, altered mitochondria, high turn-over of the distal segment of photoreceptors, the synthesis of opsins and retinal, along with a range of site-specific light-dependent activity, which will be detailed in the present review, at the border between the outer retina and inner choroid. At this site, the molecular mechanisms of light encompass the genetic activation of a number of autophagy-related genes, which are key in counteracting light-induced damage and fostering the molecular mechanisms underlying vision. As we shall see, the oxidation of proteins, lipids and sugars as well as mitochondrial alterations require a prompt autophagy-mediated clearance, which is needed to promote retinal integrity and sustain the physiology of vision. In fact, the phagocytosis mediated by microtubule-associated protein 1A (I)/1B (II) Light Chain 3 (LC3) of the outer segment of the photoreceptors allows visual acuity and prevents oxidative damage, which is triggered by light in the presence of a high oxygen extraction from the inner choroid. Light induces LC3 as well as a high number of small lysosomes, autophagosomes, and the merging between these organelles to form auto-phagolysosomes, through specific molecules which trigger autophagy at different steps [1].

### 1.2. The Occurrence of Specific Autophagy-Related Structures within Baseline and Degenerating Retina

**Figure 3:**
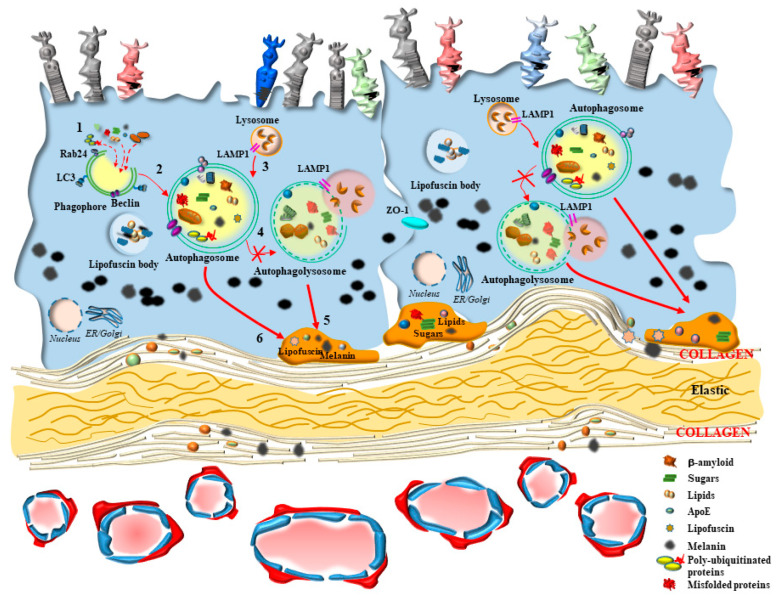
Autophagy impairment within RPE mimics most features of AMD. When autophagy is impaired selectively within RPE cells, a number of pathological effects mimicking the fine neuropathology of the early steps of AMD are induced. These include stagnant vacuoles within altered RPE cells, which modify their shape and size along with the amount of pigmentation. The autophagy pathway is already defective during the early stages when phagophore (1) should start vacuolization to proceed towards autophagosome formation (2). A defect in the merging of autophagosomes (2) and lysosomes (3) leads to a defective generation of auto-phagolysosomes (4). Nonetheless, the massive defect in lysosome clearance is in excess, compared with a decrease in auto-phagolysosome formation. Therefore, slowed autophagy flux leads to an accumulation of auto-phagolysosomes. These abundant stagnant vacuoles include lysosomes and autophagosomes, which increase their size, and persist within the cells without any further clearance, or are released towards the Bruch’s membrane to accumulate in the process of drusen formation. In fact, altered autophagosomes (5) or lysosomes (6) may be extruded through exocytosis or as myelinosomes. These processes (5, 6) are believed to generate extracellular drusen, which features non-digested substrates of the lysosomes, such as lipo-melanofuscin, lipids, sugars, misfolded proteins, including beta-amyloid, and altered mitochondria. The distal segment of RPE cells reduces cell processes, making melanin content cluster within the cell bodies, becoming scattered between distal segments of the photoreceptors. The space between RPE cells, which is normally absent due to the occurrence of abundant tight junctions, becomes evident following autophagy inhibitions due to a loss of specific tight junction proteins such as zonula occludens protein 1 (ZO1).

If one analyzes autophagy structures at this level, autophagosomes and lysosomes are small and abundant, in order to cope with the need to provide a quick cycling of these organelles intensely involved in the clearance of altered mitochondria, protein lipids and sugars, along with melanin and photo-pigments. Thus, it is not surprising that, among all retinal layers, the RPE possesses the highest autophagy rate [1]. This is fundamental in keeping steady retinal integrity during baseline conditions and mostly in counteracting retinal degeneration. Although autophagy alteration may be variably described in several types of retinal degeneration, the relevance of autophagy in the outer retina is in its coping with the site-specific pathology of age related macular degeneration (AMD), which is known to affect, firstly and mostly, the outer retina. This is why, when describing autophagy derangements in the present review, we focus on AMD. For the benefit of clarity, the main features of AMD will be described in Section 3. The relevance of autophagy within RPE cells is clearly evident in experimental models, where specific autophagy-inducing genes are knocked out [2]. This is key at the onset and during the course of genetically-induced experimental AMD. In fact, when knocking down autophagy-related genes, specifically within RPE cells, the occurrence of retinal abnormalities mimicking those described in human AMD is consistently documented [2] (Figure 3). These similarities include stagnant vacuoles within altered RPE cells, which modify their shape and size along with the amount of pigmentation, and the occurrence of frank RPE cell death (Figure 3).

These models were developed in *ATG5* and *ATG7* conditioned knock out, where gene manipulation was specifically carried out within RPE cells. Some alterations, such as neovascularization and inflammation, are rarely observed in these models. This suggests that autophagy dysfunction within RPE cells may reproduce most of the features which characterize dry AMD, although it is likely that additional structures need to be affected within the choroid/retinal border to fully develop the whole pathological hallmarks of AMD (as described in Section 3), including the wet variant. With this aim, the role of autophagy within the inner choroid deserves specific attention in order to understand mutual interactions between outer retina and inner choroid.

### 1.3. The Synergism of Autophagy in the Outer Retina/Inner Choroid

The seminal role of autophagy in sustaining retinal integrity during AMD is likely to be naturally induced by long wave-length light pulses and light-sensitive phytochemicals, which are reported to maintain retinal structure and visual acuity, as well as to counteract retinal degeneration [3,4,5,6,7,8,9,10,11,12,13,14,15,16,17,18]. In contrast, short wave-length light and metabolic dysfunctions may induce deleterious effects by altering a number of chemical species, which undergo conformational changes, deranging a number of organelles, which are affected during the high metabolic turn-over of this part of the retina. In this case, autophagy is key to counteracting the deleterious effects of light by removing damaged structures, which require a powerful and fast replacement [19]. This applies to proteins, lipids and sugars and extends to specific autophagy-dependent organelles, such as mitochondria. All these structures may represent a target of deleterious short wave-length, which may produce free radicals, altering DNA integrity, disrupting tissue structure and sustaining retinal degeneration [20,21,22,23]. In the retina, as well as in the central nervous system [24,25], autophagy activation significantly depends on the regulation of the molecular complex, mechanistic target of rapamycin (mTOR).

**Figure 4:**
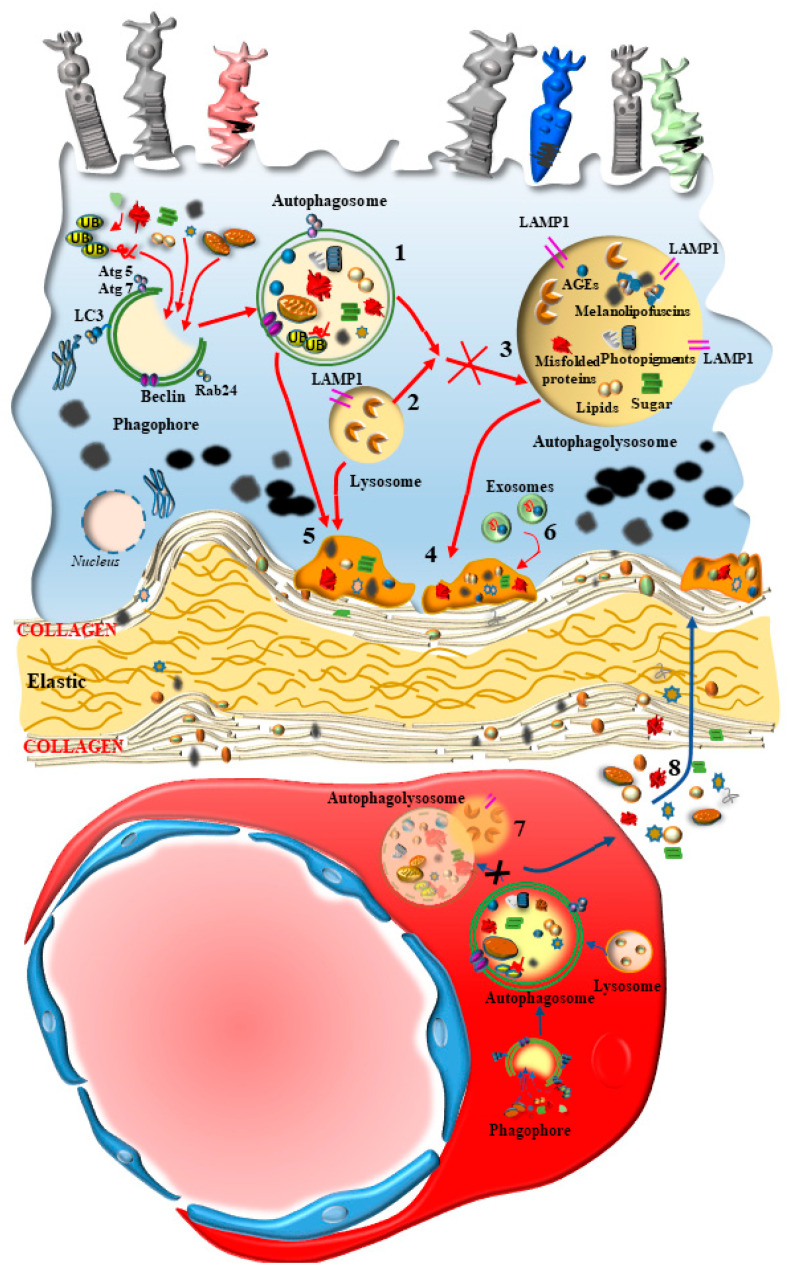
Bipolar alterations converge in altering the Bruch’s membrane (BM). The occurrence of alterations in the BM in the course of AMD may depend on converging pathological factors coming either from RPE cells and/or from the CC. In detail, the disruption of the thin collagen and elastic layers of BM may be concomitantly generated by the drusen components (described in Figure 3), which contact with and disrupt BM. Specifically, altered and stagnant autophagosomes (1) and lysosomes (2) formed in the RPE may produce stagnant and persistent intracellular RPE auto-phagolysosome vacuoles (3), being extruded towards the BM both as authentic organelles (4; 5) or upon commitment towards the exosome compartment (6) in order to deliver non-digested toxic species, which may potentially alter the structure of BM. Concomitantly, similar effects occur within the cells of CC. In fact, altered autophagy in the CC generates stagnant auto-phagolysosomes (7), which may be also extruded towards the BM (8). In this way, converging bipolar mechanisms concur to produce BM alterations. The relevance of these factors emerging from an altered autophagy within RPE, compared with those produced by an altered autophagy within CC, is likely to depend on specific isotype of AMD, where systemic risk factors are more effective in altering the autophagy status in the CC, whereas an intrinsic retinal defect is likely to be more relevant in altering RPE cells. In most cases, both sides are engaged, since systemic risk factors add to the frail autophagy equilibrium of RPE cells, which progressively deteriorates during age.

In fact, mTOR activation is generated following a number of insults, including oxidative damage, metabolic alterations, and altered blood flow. Such an activation of mTOR suppresses the level of autophagy, which is fundamental in sustaining the physiological activity at the inner choroid/outer retina border and preventing oxidative damage [24,25]. The activity of mTOR is a pivot in modulating the antioxidant activity in the retina and counteracting free radicals, which are constantly generated. In the course of retinal degeneration, mTOR is known to sustain retinal pathology. In fact, mTOR is altered within RPE and inner choroid in the course of AMD [26]. In detail, the regulation of autophagy activity by mTOR appears to be orchestrated in synergy with the choroid domain (choriocapillaris, CC) and retinal border (RPE) [26]. In this way, the autophagy status would produce simultaneous and synergistic effects on both sides of the Bruch’s membrane (BM) under the modulation of mTOR. When autophagy-mediated enzymatic clearance of altered organelles is engulfed by an excess of substrates, RPE cells may use an alternative pathway to clear non-digested material by promoting cellular extrusion (Figure 3). In this way, polymorphous inert aggregates within RPE may be released as myelinosomes, recently described by Yefimova [27]. In fact, myelinosomes add to lysosomes, autophagosomes, and exosomes in the RPE of the retina in order to provide proteo-stasis and clearance of damaged organelles, which is seminal in maintaining retinal integrity, as described by Ferrington et al. [28]. This metabolic polarization of RPE cells extrudes material towards the BM, which separates the outer retina from the inner choroid. This alters the permeability and morphology of the BM. In fact, during AMD as well as the experimental models developing AMD, a thickening of the BM is described [29]. These authors demonstrated that thickening of BM is concomitant with a decrease in autophagy activity within RPE cells, as shown by alterations of LC3 and Lysosomal-Associated Membrane Protein 1 (LAMP1) labeling. These cells develop an altered LC3-II/LC3-I ratio as an index of an impaired autophagy flux, which is concomitant with an altered amount of specific autophagy-related proteins [29]. One may consider the alterations of the BM as the mere consequence of altered autophagy within RPE cells. However, in persons affected by AMD, alterations of the BM are associated also with a defective autophagy in the contiguous inner choroid at the level of the pericytes and endothelial cells within CC [30]. Pericytes alterations are presently underscored, since recent evidence shows that they may anticipate alterations within endothelial cells of the CC. In detail, when pericytes are dysfunctional, the degenerating inner choroid produces massive vesicular and fibrillary aggregated materials moving towards the BM [31,32]. Thus, it appears that a thickening of the BM in the course of AMD may indeed be the consequence of both an external perturbation, coming from the inner choroid (CC), and an internal alteration, coming from the outer retina (RPE) (Figure 4).

Thus, an orchestrated defect of clearing mechanisms placed at the choroid–retinal border is likely to produce a deleterious synergism in the course of AMD, fostering the accumulation of altered mitochondria, photo-pigments, melanin, sugars, lipids and proteins to form lipofuscin, and their accumulation into drusen. In fact, in the presence of altered clearance, the enhancement of drusen formation between the BM and RPE can be observed [33,34,35].

The contribution of various clearing pathways within various structures of the choroid–retinal border is analyzed here. This review focuses on the functional interactions which take place at the anatomical border between choroid and retina, where a dysfunctional RPE may perturb CC and vice-versa, under the effects of a site-specific defect of autophagy. This pathway, working at the border between inner choroid and outer retina, defines and regulates a sort of neurovascular unit. This anatomical complex is key for autophagy to maintain anatomical integrity and visual processing while, in AMD, it represents the pathological unit driving retinal degeneration.

## 2. Interdependency of Various Targets of Autophagy within RPE and CC in the Retinal Neurovascular Unit

**Figure 5:**
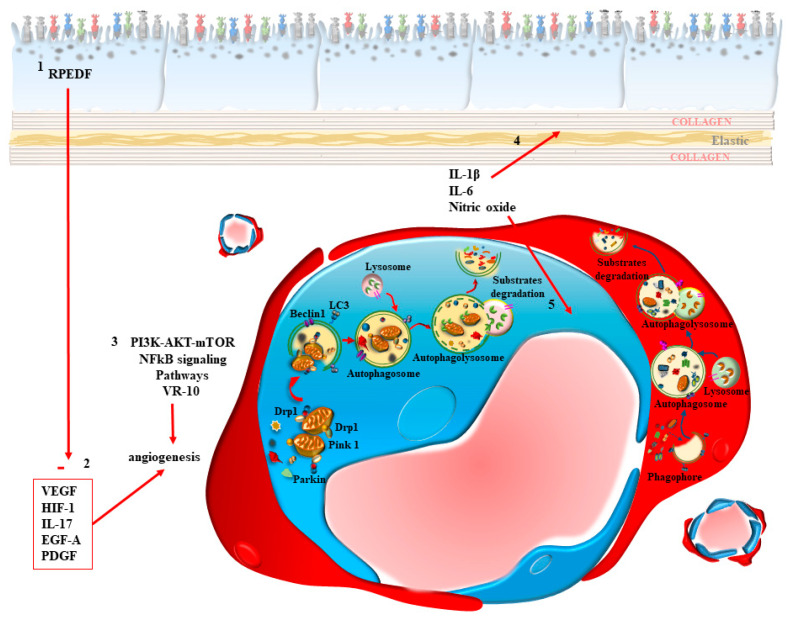
Within CC, autophagy is key in both endothelial cells and pericytes. Within CC, an altered autophagy status leads to accumulation of dysfunctional mitochondria, where the mitophagy proteins, Dynamin-related protein 1 (Drp1), PTEN-induced putative kinase1 (PINK1) and Parkin, no longer interact to promote mitochondrial clearance within nascent autophagosomes. A defect of autophagosome formation and a failure in their progression towards lysosomes generates a defect in the clearance of a number of organelles and chemical species. Analogous effects involve the pericytes, where these alterations appear to anticipate the dysfunction of endothelial cells. These phenomena, apart from producing damage to the BM due to the accumulation of non-digested material at the choroid side of the BM itself, produce the synthesis of a number of compounds, which exert a powerful angiogenetic effect. As we shall summarize in Section 3, defective autophagy in the CC exerts a prominent role in causing AMD in the course of systemic disorders such as diabetes, hypercholesterolemia, metabolic syndrome, and other specific metabolic disorders. Nonetheless, the dysfunctional CC may be induced by primary alteration within RPE cells. In fact, alterations of RPE may produce trophic factors stimulating angiogenesis, such as Vascular Endothelial Growth Factor (VEGF) and Platelet-Derived Growth Factor (PDGF), in the course of retinal degeneration by acting on the CC to induce endothelial cells and pericyte proliferation, migration and angiogenesis, in order to shift AMD from a dry into a wet variant (refer to Section 3 for defining these AMD isotypes). Similarly, a precursor of Epidermal Growth Factor may support angiogenesis via phosphatidylinositol 3-kinase (PI3K)/protein kinase B (AKT)-mTOR and Nuclear Factor kappa-light-chain-enhancer of activated B cell (NF-κB) signaling pathways. Similarly, the expression of VEGF may derive from endothelial cells to stimulate angiogenesis via action on the AKT/mTOR pathway. It is remarkable that healthy RPE cells may counteract these angiogenetic effects by releasing retinal epithelial pigment-derived factor (REPDF), which inhibits the very same angiogenetic pathway. The crosstalk between CC and RPE may also act in the opposite direction, from the CC to the RPE. In fact, upon autophagy inhibition, CC cells may synthesize interleukins such as IL-1β, and IL-6 along with nitric oxide. These molecules may activate the inflammasome focally within CC although, when AMD shifts towards the wet form, they migrate through the BM to activate macrophages passing through newly generated blood vessels in the disrupted outer retina.

The autophagy occurring within endothelial cells of the CC is seminal in sustaining the integrity of the retina (Figure 4). In fact, protein and mostly mitochondrial removal within CC endothelial cells counteracts ongoing injuries. In keeping with autophagy, the removal of altered mitochondria from the CC through mitophagy is critical to prevent and counteract retinal degeneration. This occurs by preventing the fragmentation of stagnant damaged mitochondria, which instead are removed by an effective mitophagy. In this way, the microvascular damage in the retina is prevented [36] (Figure 5). Thus, the inner choroid represented by the BM and the CC contributes to sustaining the integrity of the outer retina.

In detail, the physiological role of the CC is bound to the presence of appropriate blood vessels to maintain the viability and the high metabolic rate of RPE cells. The ability to produce these effects is bound to the integrity and permeability of the BM. In this way, the inner choroid and outer retina work in symbiosis to provide anatomical integrity for the retina and to sustain visual function. According to Edwards and Lutty [37], a functional unit can be described, which is composed of the following structures: (i) outer segment of photoreceptors; (ii) RPE cells; (iii) BM; (iv) CC. It appears that these structures, which are orchestrated by specific metabolic pathways, need to work as a single physiological unit, which loses its function in the course of retinal damage, and mostly in the course of AMD. In fact, during AMD, the CC loses its function and integrity early [37]. Cai et al. [38] described how a combined defect of the autophagy stimulator microRNA-29 within RPE and the choroid vessels occurs in AMD. This may lead to autophagy inhibition concomitantly in the inner choroid and outer retina. Autophagy inhibition is known to promote neovascularization starting from CC under the effects of inflammasome activation within macrophages, which can be induced by Interleukin-1beta (IL-1β), IL-6 and nitrite oxide [39,40]. This is why, in baseline conditions, mTOR dependent autophagy activity in the CC is crucial to modulate the permeability for specific molecules, which may or may not cross the CC to reach the outer retina. In addition, autophagy modulates angiogenesis in the retinal choroid border [41]. Although so far most manuscripts attribute these roles of the CC to endothelial cells, mounting evidence is now demonstrating the seminal role of pericytes and the engagement of this cell type in the early stages of AMD as the first cell responsible for the alterations occurring in the CC, when endothelial cells are not yet involved. Each cell type of the CC will be analyzed separately, at first in baseline and general pathological conditions and, starting from Section 3, specifically in the course of AMD.

### 2.1. A Focus on RPE

The RPE cells possess the highest autophagy rate in the whole retina [1]. In fact, autophagy in the RPE is key in removing a number of structures, including various chemical species and subcellular organelles. Most proteins and lipid clearance is autophagy-dependent [19,21,42]. This is confirmed by the fact that autophagy defects lead to accumulation of misfolded proteins and lipid droplets. This extends to cell organelles, since autophagy failure alters the structures and amount of various organelles within RPE [21,43,44,45]. In particular, when autophagy progression does not occur properly, abnormal accumulation of misfolded proteins, lipids and organelles is evident within giant lysosomes [46]. Here, proteins are prone to bind sugars to produce advanced glycation end products (AGEs) and lipids, in order to generate lipofuscin [33,47,48,49], which may further bind melanosomes to form lipo-melano-fuscin [50]. These structures, including mitochondria, represent cellular debris, which produce the building blocks of drusen [40,51]. In fact, these aggregates may be already evident within lysosomes and accumulate in high amounts within the border between RPE and BM [46]. Thus, the physiological role of autophagy within RPE is connected to innumerous functions exerted by these active cells. In line with what is reported above, the altered chemical species which are generated in baseline conditions by the powerful oxidizing effects of light can be tolerated pending an autophagy process [21,52,53,54,55,56]. Similarly, the oxidative metabolism which characterize these cells needs to be supported by an intense turn-over of mitochondria [57,58,59,60]. Again, the need to clear a variety of chemical species requires the support of a massive number of active lysosomes [61,62,63]. The intense generation of structures to be metabolized requires the generation of ceiled, a segregated cell compartment to harvest this obsolete material for delivery to enzymatic clearance. This explains why a high number of autophagosomes are generated during short time intervals [1,19,29,63]. This is magnified by the specific role of RPE cells in coping with the fate of the outer segment of photoreceptors [64,65,66]. In fact, the phagocytosis of the outer segment of cones and rods occurs according to a quite unique process, LC3-associated phagocytosis (LAP) [67], where phagocytosis takes place within vacuoles, which are further stained with LC3 to generate a type of photoreceptor-dedicated autophagosome. This is supposed to enhance the classic phagocytosis, allowing a faster and massive turn-over of the photosensitive domains of photoreceptors. In this way, such a dedicated autophagy/phagocytosis, i.e., LAP, is seminal in sustaining the cell biochemistry in the physiology of vision. The process of LAP, which is specific for RPE cells in the choroid/retinal border, is aimed at ingesting the distal disk membranes of cones and rods to provide their renewal, in order to keep vision effective. The biochemical steps in LAP need to regulate the activity of a number of molecules within the retinal pigment epithelium (RPE), such as melanoregulin [68], rubicon and epidermal growth factor receptor (EGFR) [69].

To witness the relevance of LAP in the baseline activity of RPE cells, upon autophagy during AMD, drusen occurring between RPE cells and the BM features photopigment residues [70]. One may assume that drusen’s structure somehow witnesses the various roles of autophagy within RPE cells. Within the drusen, pieces of endoplasmic reticulum occur, along with melanosomes [19]. Again, the structure of lipofuscin, which occurs abundantly within AMD-RPE cells and within drusen in the course of AMD, is consistent with their main origin from RPE cells. In fact, they contain a number of lipids, sugars, and proteins, along with photopigments residues and altered cell organelles. The fate of lipofuscin, which lacks appropriate digestion, is to engulf persistently giant lysosomes within AMD-RPE cells. Therefore, most are released vie exosomes to be hosted in the sub-choroidal space, being recruited to form drusen [33,71,72].

### 2.2. A Focus on Endothelial Cells

The viability of choroid–retinal endothelial cells is profoundly affected by the autophagy status, both in baseline conditions and during systemic metabolic disorders, such as diabetes [41] or familial hypercholesterolemia, which may trigger AMD due to increased sugars and lipoproteins, producing damage to endothelial cells through autophagy inhibition, which in turn can be rescued by autophagy activators [73]. It is not surprising that, in the course of these metabolic disorders, within CC the autophagy status is altered within endothelial cells, as shown in most vascular districts of the body (Figure 5). Autophagy dysfunction alters the mitochondrial status and the lysosomal activity within endothelial cells [73]. Specifically, endothelial cells of the inner choroid (CC) are rescued by autophagy activation, as shown by Zhang et al. [36]. These authors provided evidence showing that endothelial cells, during retinal degeneration, undergo autophagy suppression, which inhibits mitochondrial removal and lipidation of LC3II and p62 degradation. These effects can be replicated by the autophagy inhibitor 3-methyladenine (3-MA), while they are counteracted by the autophagy activator rapamycin [36]. In addition, the specific autophagy inhibitor 3-MA is deleterious for endothelial cells, since it worsens the damage induced by high glucose levels, which explains why diabetes, by causing an autophagy-dependent damage to the CC, may foster the onset of AMD. Consistently, the mTOR inhibitor and autophagy activator rapamycin is able to rescue the endothelial damage in the choroid retinal vessels [36]. The mitochondrial removal is known to be activated by the autophagy-dependent protein PTEN-induced putative kinase1 (PINK1) and parkin. In fact, a strong trigger for mitochondrial removal is produced by the PINK1/parkin interaction at mitochondrial level [74]. Such an interaction is impeded during retinal degeneration. This is why the silencing of PINK1 aggravates retinal damage. This is in line with a defective amount of endothelial Dynamin-related protein 1 (Drp1), which promotes mitochondrial fission and mitophagy, during retinal degeneration. In diabetes, Drp1 is detached from mitochondria and it is no longer able to promote mitophagy, due to an excess of hexokinase II (HK-II), which removes Drp1 from mitochondria and prevents its interaction with PINK1 to trigger mitophagy [36]. It is remarkable that alterations in newly formed blood vessels, which may be induced in the retina by systemic administration of the diabetes-inducing compound streptozocin, mimics those occurring in AMD, such as vascular leakage and abnormal newly-formed blood vessels. These alterations can be reversed by the autophagy activator rapamycin [36]. Alterations in the CC may also derive from specific molecules released from neighboring RPE cells. In line with this, it is well-known that trophic factors stimulating angiogenesis, such as Vascular Endothelial Growth Factor (VEGF) and Platelet-Derived Growth Factor (PDGF), are produced in the course of retinal degeneration by RPE cells when a shift from dry to wet form of AMD takes place. The release of VEGF and PDGF from RPE cells is inhibited by autophagy activators such as rapalogs, which depress the primary transcript of these proteins, which in turn would induce endothelial cells towards increased proliferation, migration and angiogenesis [75]. This is confirmed by using the natural autophagy activator resveratrol, which inhibits Epidermal Growth Factor precursor homology domain A (EGF-A) via suppression of phosphatidylinositol 3-kinase (PI3K)/protein kinase B (AKT)-mTOR and Nuclear Factor kappa-light-chain-enhancer of activated B cell (NF-κB) signaling pathways. In detail, resveratrol inhibits the phosphorylation of the inhibitor of nuclear factor kappa B (IκB), which inhibits NF-κB [76]. In this way NF-κB cannot migrate any longer to the nucleus, where it stimulates the transcription of VEGF-A [76]. Similarly, the natural autophagy activator berberin inhibits the expression of VEGF from retinal endothelial cells via its action on the AKT/mTOR pathway [77].

### 2.3. A Focus on Pericytes

The powerful effects of autophagy in suppressing the proliferation and migration of endothelial cells is backed up by analogous effects on pericytes, which are present in the CC and proliferate also in AMD. In fact, the failure of anti-VEGF based therapy is partially due to the residual activity of proliferating pericytes, as hypothesized by Asani et al. [78] (Figure 5). Although being missed out for a long time, pericytes are seminal in the course of AMD, considering mainly the wet variant and AMD induced by systemic metabolic disorders. In fact, the mTOR inhibitor and autophagy inducer rapamycin exerts a powerful effect in suppressing the amount of pericytes, and inhibits pericytes’ proliferation in the CC. These mechanisms occlude neovascularization from the choroid to the outer retina, which otherwise occurs in the course of wet AMD (Figure 5). In fact, the activity of mTOR is increased in order to suppress autophagy in the course of neovascularization within wet AMD [79]. The anti-angiogenic effects of autophagy induction are presently under investigation to design novel compounds, which may be useful in suppressing the excess of angiogenesis occurring in wet AMD. This is the case for the thrombospondin-1 synthetic peptide VR-10, which increases the LC3II/LC3I ratio and degrades p62, being a powerful autophagy inducer [80]. This peptide upregulates the anti-angiogenetic factor Retinal Pigment Epithelium-Derived Factor (RPEDF), while downregulating pro-angiogenetic factors (VEGF, HIF-1 and IL-17) [80]. In line with autophagy-based treatments of abnormal genesis of blood vessels, a number of molecules acting as autophagy inducers are currently under evaluation in the course of choroidal neovascularization [81]. The analysis of CC in relationship with its effects on the autophagy machinery in the course of AMD naturally drives attention to the role of this clearing pathway within endothelial cells. This is naturally due to the development of endothelial cell proliferation in the course of neovascularization, which occurs in the inner choroid to surpass the BM and invasion of the outer retina, when AMD shifts to the wet variant. However, as previously mentioned, in addition to endothelial cells, pericytes promote neovascularization, through a significant role in the blood vessels of the CC (Figure 5). So far, pericytes have been poorly investigated in AMD and are impaired, even when the damage to endothelial cells is not yet evident. This is why, in the present review, a special focus is given to these cells.

**Figure 6:**
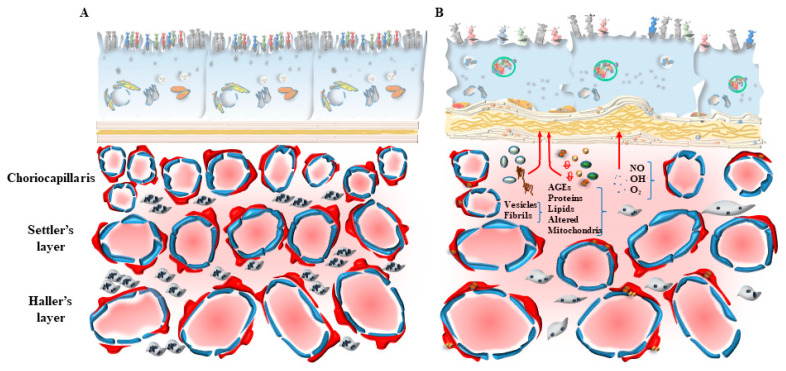
The autophagy pathway in the normal (**A**) and diseased (**B**) choriocapillaris. An autophagy defect in the CC is primarily sensed by pericytes, which feature early occurrence of undigested autophagy substrates, witnessing to a defective metabolism of Advanced Glycation End products (AGEs), proteins and lipids. These AGEs are found to be abundant around pericytes within choroid debris. This is concomitant with a loss of intermediate filaments from pericytes and mitochondrial alterations. These phenomena are associated with a defective autophagy machinery. In fact, within pericytes, the abundance of intermediate filaments is related to the presence of effective autophagy, which confers protection to this cell type. The vulnerability of pericytes in AMD may be the consequence of degenerating choroid melanized stromal cells (lamina fusca), which worsens the impact of light- or systemic-induced free radicals.

Pericytes are placed in the CC to surround externally the fenestrated endothelial cells sharing the same basal lamina (Figure 5). The inherent structure of the CC features a considerable defect in the number of pericytes, since they do not ceil fully the perimeter of the capillaries, while an absence of pericytes is noted externally in endothelial cells. This may be a natural vulnus in the anatomy of the CC blood vessels, which fosters the development of AMD. In fact, drusen deposits, apart from aggregating just below the membrane of RPE, can be detected across the BM among inner collagen fibers to form the linear deposits described by Curcio and Millican [82] (Figure 4). The occurrence of deposits in the form of vesicles and fibrils is evident in aged inner choroid, which poses the question as to whether an extended distribution of extracellular aggregates external to the BM takes place in the genesis of AMD. The role of abnormal perycites was postulated in the progression of this condition. In a recent paper, Nag and colleagues described the occurrence of alterations in pericytes in the course of neovascularization [30]. In these conditions, pericytes feature dark mitochondria and cell processes in touch with debris at the level of the basal membrane close to BM. At this stage, pericytes lose their basal lamina and intermediate filaments, even when the endothelial cells are still intact. In this way, two domains can be identified at the choroid/retinal border where debris and aggregates can be formed (Figure 4): the classic domain, just beneath the RPE, and the other domain within the choroid, which is supposed to proceed through the BM in the wide space between outer collagen fibers in order to disarrange the elastic lamina (Figure 4). It is postulated that these extra-cellular aggregates may be the earliest event in altering the morphology of pericytes within the CC. Alternatively, due to the close space between pericytes and early choroid aggregates, it may be postulated that a metabolic defect at the level of the pericytes may be responsible for extracellular accumulation of vesicles and fibrils in the choroid. In fact, early occurrence of undigested autophagy substrates within pericytes may witness a defective metabolism of AGEs, proteins and lipids (Figure 6). These AGEs are found to be abundant around pericytes within choroid debris. This is concomitant with a loss of intermediate filaments from pericytes and mitochondrial alterations. These phenomena are associated with a defective autophagy machinery. In fact, within pericytes, the abundance of intermediate filaments is related to the presence of effective autophagy, which confers protection to this cell type [30]. These findings suggest that even subtle alterations of pericytes, under the effects of a defective autophagy, may promote endothelial alterations, which develop progressively during the disease course. The vulnerability of pericytes in AMD may also be the consequence of degenerating choroid melanized stromal cells (lamina fusca), which loses their melanin content during age, making more deleterious the impact of free radicals formed either by a greater diffusion of ultraviolet quanta during light exposure or from circulating oxidative species. These extracellular factors add to systemic metabolic disorders and specific autophagy-dependent intra-cellular defects of pericytes in the CC. In fact, pericytes in baseline conditions feature a lower amount of ongoing autophagy machinery compared with contiguous endothelial cells [30]. This may explain why, within CC, pericyte degeneration may precede alterations in endothelial cells in the course of AMD. In line with a selective autophagy defect, alterations occur early within CC pericytes during AMD (as shown by representative Figure 6).

### 2.4. A Focus on BM

In summary, the relevance of autophagy in the inner choroid in regulating the integrity and pathology of retinal degeneration remains largely to be investigated. Even the BM is affected by autophagy impairment since, upon autophagy inhibition, the BM undergoes progressive thickening [29]. However, it remains unexplored as to whether such an effect is indirect, due to a leakage of substrates from the CC and/or the RPE (Figure 4), or the structure of the BM is specifically and directly regulated by the autophagy machinery. The BM alterations which are typical of AMD are mostly related to the impaired autophagy in the CC at the level of both pericytes and endothelial cells [30]. This is confirmed by the onset of a specific disorder of the CC, where increased angiogenesis occurs along with altered structure of the BM [83].

Despite an increasing number of investigations, the regulation of autophagy in the inner choroid remains largely unexplored and deserves further studies. When considering the most internal membrane classified as part of the choroid, the BM, evidence is provided that autophagy reverts the thickening of the BM [29]. During retinal degeneration the dysfunction of the BM appears to be closely related to autophagy-dependent alterations within pericytes and endothelial cells of CC [30]. In fact, a rare disorder of the CC, with increased angiogenesis and bleeding, produces macular drusen deposition [83]. The data produced so far concerning the impairment of the BM during autophagy inhibition are still in need of further investigation [50]. In fact, to our knowledge, no specific experimental effort has been carried out to measure how autophagy may alter the synthesis and release of collagen fibers from fibroblasts involved in the integrity of BM. Indirect evidence suggests that, autophagy being a pathway which counteracts fibrosis, a hypothesis to be tested consists in specific anti-collagenogenic effects of autophagy activation [84,85], which would further clarify why, in the course of AMD and/or autophagy inhibition, a thickening of the BM takes place in which the elastic component is reduced, compared to altered collagen structure which is increased. In fact it is demonstrated that, during autophagy inhibition, collagen structure is stimulated, while the elastic fibers are suppressed [86]. This is evident in the skin [84] upon blue and UV light exposure. The cells, where autophagy modulates the synthesis of collagen and elastic fibers, correspond to fibroblasts [84,86]. These fibroblasts occur in the retinal choroid border and are known as choroidal mesenchymal fibroblasts, which are connected with RPE cells through paracrine signaling [87].

## 3. Dysfunctional Autophagy in the Inner Choroid/Outer Retina Is Mostly Relevant for AMD

Age-related macular degeneration (AMD) represents the most common cause of blindness in the Western World [51,88,89,90]. The prevalence of such a disorder has been dramatically increasing in the last decades [91]. Retinal degeneration may occur in different areas (geographic degeneration), although the recruitment of the macular region is essential. The pathology of AMD consists of two main variants named dry and wet. In the dry variant, solid drusen aggregates prevail, while in the wet variant, soft drusen occurs in the context of retinal exudates and newly formed blood vessels [51]. Sometimes the wet variant takes over the dry form over time, while in some cases AMD presents suddenly in the wet isoform. The dry variant is slower, though invariably leads to deterioration and ultimately blindness. In fact, at clinical level the disease manifests with a loss of visual acuity and contrast sensitivity, which are provided by a selective impairment of macular vision [51,90,92,93]. A typical symptom often described in AMD consists of distorted, wavy lines along the horizontal and vertical axis known as metamorphopsia [93]. The mechanisms which produce a loss of visual acuity are considered to rely on the loss of retinal planarity and a loss of foveal cones, which also suppresses contrast sensitivity. A massive loss of photoreceptor activity is eventually responsible for these symptoms [67]. However, it is very likely that an impairment of visual acuity may occur early on, when the integrity of photo-receptors is fully preserved. This is likely to depend on the failure in the biochemical cascades generating the physiology of vision. In fact, the massive involvement of an autophagy failure in the physiopathology of AMD is supposed to alter, at first, the turn-over of photo-pigments [67]. The relevance of a number of disorders in the genesis of AMD is reported. In fact, retinal specific autophagy alterations [94,95], blue light-induced autophagy failure leading to an excess of oxidized substrates [52,54,59,96,97,98,99,100,101], and systemic metabolic disorders suppressing retinal autophagy, such as diabetes [102,103] and familial dyslipidemia [104,105], are detrimental in AMD. It is established that a site-specific autophagy suppression in the retina leads to AMD. Specifically, the site of the retina where autophagy is fundamental in preventing AMD is the outer retina and this area extends to the inner choroid, passing through the BM [1,95]. The specific cells involved were previously analyzed and consist of outer photoreceptors, RPE, endothelial cells and pericytes of CC, and very likely scattered fibroblasts in the BM. Since the relevance of RPE for the disease is established at large, in the present manuscript we will start by first analyzing the role of the inner choroid in AMD, since novel findings are posing specific choroid cells and mechanisms as relevant to the generation of AMD [30,106,107]. In particular, a dysfunctional autophagy in the choroid may be responsible for delivering abnormal substrates towards the outer retina and may instigate the deposition of drusen on the outer aspect of the BM [50,84,85,86,87].

## 4. Dysfunctional Autophagy within CC in AMD

This may explain why polymorphous aggregates may develop on both sides of BM in advanced stages of AMD. In fact, an updated perspective considers drusen, beyond the protein and specifically beta-amyloid content, as a complex aggregate of lipid droplets, sugars, melanin, lipofuscin, chromatin vesicles (spheroidal structures that are made of extra-nuclear DNA leaking out of the nucleus), degenerated melanosomes, degenerated mitochondria, aqueous vesicles, and ultrafine cellular fragments. These drusen are expected to disrupt the BM causing collagen degeneration and loss of elastin, which in turn produces a loss of anatomical integrity. This allows drusen content and cellular debris from necrotic RPE cells to move across BM from the retina towards the choroid [106]. The scenario emerging from these data leads to consideration of the option that, in the course of AMD, extra-cellular material may accumulate de novo within the choroid side of the BM. In fact, when considering the etiology of AMD, diabetes and familial hypercholesterolemia are strong predisposing factors. In these conditions, a primary autophagy defect within retinal structures may not be present; however, a defective autophagy may rather depend on extra-retinal compounds which reach up the choroid-retinal border through the blood stream, within the most intimate choroid layer, the CC, which is expected to undergo the primary metabolic dysfunction triggering retinal degeneration. In these conditions, circulating extra-cellular chemical species may trigger the impairment of autophagy, which initiates only in the CC, the location to which the blood delivers these compounds. In line with this, the occurrence of Low Density Lipoproteins (LDL) within CC endothelial cells is key in regulating the autophagy pathway. In fact, as shown by Torisu et al. [107], baseline endothelial autophagy is required to maintain vascular lipid homeostasis. In detail, while low levels of LDL produce a stimulation of the autophagy flux, when LDL is in excess, autophagy structures are engulfed. This phenomenon is worsened when endothelial cells display a decreased expression of the autophagy gene ATG 7, which demonstrates how important is autophagy activity within endothelial cells in handling properly an excess of circulating lipids. When this process is impaired, autophagy substrates recruit the neighboring RPE cells [107].

In fact, Chang et al. [108] found that non-saturated lipid chains. such as oleic acid, trigger specific angiogenic factors, such as VEGF and basic Fibroblast Growth Factor (bFGF), along with stimulating AMP-activated protein kinase (AMPK)/mTOR/Ribosomal protein S6 kinase beta-1 (p70S6K), signaling and inducing hyperphosphorylation of the mitogen-activated protein kinase (MAPK) pathway, such as Extracellular signal-Regulated Kinase (ERK), Jun N-terminal Kinase (JNK), and p38 MAPK, as well as NF-κB activation. All these events are implicated in choroid neovascularization, which occurs in wet AMD. Similarly, in this condition, other AMD reminiscent abnormalities, such as basal laminar deposits, thickening of BM with drusen-containing deposits, RPE and photoreceptor degeneration, are described following a high fat diet in a mouse model heterozygous for the Peroxisome proliferator-activated receptor Gamma Coactivator 1-alpha (Pgc-1α). These Pgc-1α+/- mice following a high-fat diet undergo autophagy suppression with decreased mitochondrial activity and increased levels of reactive oxygen species and inflammatory response in the retina [109]. Even in this context, the interaction of choroid-derived blood constituents with RPE cells is critical. For instance, co-culturing damaged RPE with blood-derived macrophages leads macrophages to release pro-angiogenic factors through the activation of inflammasome [39].

## 5. The Loss of Autophagy within RPE Cells in AMD

Despite already evidenced in the previous part, where the role of the massive engagement of autophagy was evidenced, even in baseline conditions within RPE cells., the classic hallmark of AMD consists of altered RPE cells, where an early and severe defect in the autophagy machinery is well-documented. In fact, in baseline conditions, RPE cells undergo an excess of metabolic activity due to their multiple functions. Namely, most of the autophagy machinery in the RPE is dedicated to the removal of the outer segment of photoreceptors [110].

The engulfment of the autophagy process in the RPE occurs when autophagy is inhibited. This occurs when lysosomes cannot degrade metabolic by-products. This condition is typical of AMD at the early stages, where stagnant lysosomes are present in excess, containing non-digested material in the form of lipofuscins or lipo-melano-fuscin [50]. In fact, lipofuscin blocks lysosomal enzymes, constraining these organelles to release their content below the plasma membrane, where this material aggregates to form drusen. Concrete evidence of a dysfunction of autophagy in AMD is obtained through the isolation of RPE cells from donor patients affected by AMD, compared with RPE cells from controls. When compared with controls, RPE cells from AMD patients possess classic alterations [50], which can be related to the impairment of autophagy. The autophagy flux is indeed impaired in AMD-RPE cells compared with controls, at least concerning lysosomes, which are stagnant and dilated, as shown by large LAMP1 positive spots present in a high amount compared with small LAMP1 positive puncta detected in control RPE cells. In detail, as reported by Golestaneh et al. [111], immunostaining for LAMP1 shows enlarged LAMP1-positive structures in AMD-RPE compared with small discrete puncta observed in normal RPE. This is concomitant with depressed autophagy, as measured by the ratio of LC3II/LC3I. The slowed autophagy flux occurs in turn, along with a relented decrease of p62, even following autophagy stimulation (through starvation). This slowed autophagy flux may be responsible for accumulation of lipid droplets and glycogen granules within AMD-RPE cells, which in turn may lead to drusen formation. In fact, in a manner which is reminiscent of inclusions occurring in autophagy-dependent neurodegenerative disorders, drusen structure in AMD is composed of several autophagy-dependent proteins and organelles [112].

The occurrence of autophagy dysfunction within RPE from AMD patients emerges in several studies carried out in the last few years [42,56,95]. The accumulation of autophagy proteins along with autophagy substrates and exosomes within drusen suggests a potential extrusion of altered organelles, lipids, sugars and proteins [42].

An autophagy defect within RPE may lead to a number of downstream deleterious events. In fact, altered organelles and misfolded proteins, sugars and lipids are expected to trigger direct toxicity in the cell. This is likely to occur at short time intervals. The late fate of these aggregates is more questionable, since a number of reports range from a detrimental mechanical effect to an inert role as innocent bystanders, simply witnessing a previous dynamic of the disease.

## 6. An Autophagy Defect within RPE May Early Induce a Visual Defect Rather than Later Neuropathology

Thus, it is likely that a loss of the primary metabolic function, which is bound to an autophagy defect, is expressed in the disease generation more as a dysfunctional molecular defect (visual defect) rather than a morphological aberrancy (neuropathology). In this way, misfolded proteins or dysfunctional mitochondria are likely to exert their toxicity before being encapsulated within lysosomes or drusen, at early stages, when they are still playing a role in the visual processing, being free in the cytosol before their aggregation. This hypothesis contradicts the common belief that an autophagy defect is considered not dangerous at the early stages, but only later when the volume of aggregates of autophagy-dependent structures is noticeable. According to the classic perspective, within the retina, this volume effect is commonly expected to alter the planar arrangement of the photoreceptors. This was commonly considered to produce visual distortion, such as metamorphopsia, which is quite typical in AMD [17,93]. In this way, visual loss was thought to be a secondary event solely generated by the loss of retinal planarity. If this is correct, one should expect that the measurement of the drusenoid area, calculated in the retina in the course of AMD, should predict the amount of loss in visual acuity and the occurrence of visual distortions. However, when the drusenoid area is calculated in AMD patients, a dissociation emerges between the amount of the drusenoid area and the severity of visual symptoms ([93] and representative data in Table 1). These findings lead to the hypothesis that an autophagy defect impairs visual function more as a result of a fine molecular deficit impairing the molecular mechanisms of visions, rather than a gross mechanical alteration of retinal planarity due to sub-retinal aggregates. In line with this, recent data indicate that suppression of visual acuity in AMD is the consequence of pure metabolic effects, which may be restored by autophagy stimulators [10,17,40,42,58,93]. These effects are quite independent from the amount of drusenoid deposition [93] (see also Table 1, showing the discrepancy between visual acuity, visual distortion, retinal thickness and drusenoid area).

As extensively reported in a previous manuscript [93] and in Table 1, with representative original data from two patients, the discrepancy between the loss of visual acuity and the amount of the drusenoid area is evident. Table 1 reports opposite conditions. In detail, patient 1 is seriously affected by drusen deposition, which occurs in most of the macular surface (drusenoid area 22.00 mm^2^) and surpasses by 17-fold the drusenoid area measured in patient 2 (1.32 mm^2^). Remarkably, such a serious drusenoid involvement in patient 1 occurs along with better visual acuity and less severe metamorphopsia compared with patient 2, affected by a mild drusenoid involvement. The drusenoid area represents the best index to express quantitatively the amount of retina filled with drusen. This index is calculated by multiplying the drusen number and each drusen size. This measurement is more accurate than the simple drusen number or drusen size per se, or a subjective drusen scoring system as extensively discussed previously [93]. The central thickness obtained at retinal topography provides an indirect measurement of the loss of planar arrangement of the retina. Metamorphopsia (linear distortion) is measured as the degree of loss of linear alignment within the horizontal and/or vertical axis.

## 7. The Novel Concept of “Inner Choroid/Outer Retina Neurovascular Unit”

### 7.1. Connecting Photoreceptor Stimulation with Metabolic Activity and Blood Supply

Molecular events altering vision may also occur in cell types beyond RPE. In fact, the occurrence of autophagy involves altogether the inner choroid and outer retina. This is supposed to affect an anatomical unit, which is coupled to match photoreception with metabolic requests and blood supply, as originally postulated by Spraul et al. [50].

**Figure 7:**
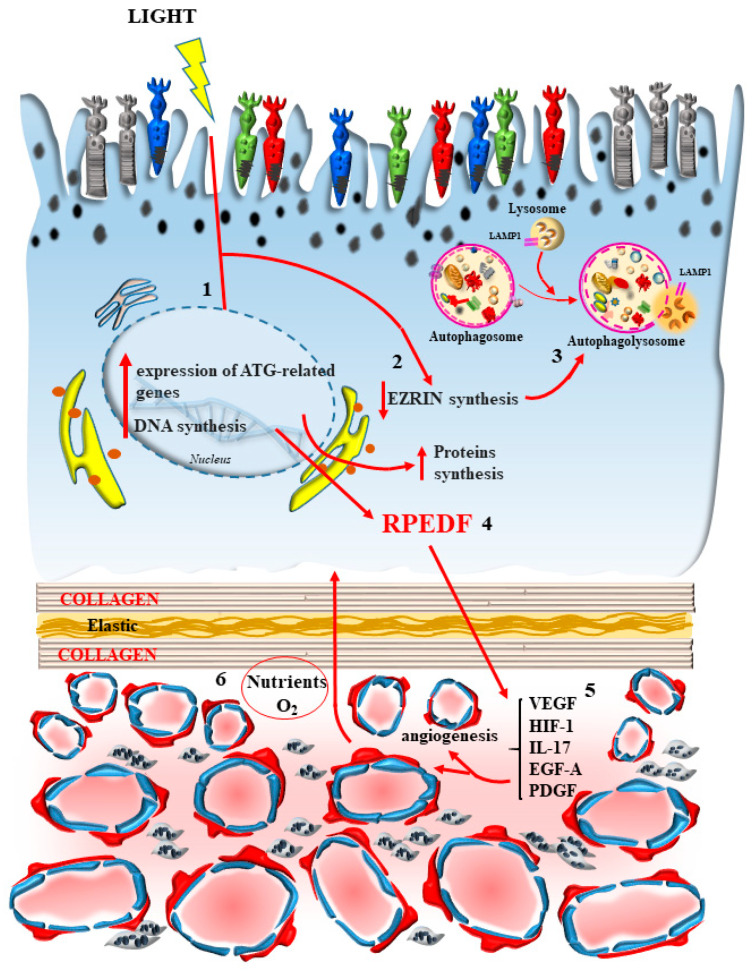
The inner choroid/outer retina unit is similar to the neurovascular unit recently described in the CNS. In line with this, in the retina, the rate of autophagy within rods varies according to the light cycle, diurnally regulated. This occurs according to the metabolic requests induced by vision. In this way, it is possible to define a retinal neurovascular unit, where the activity of the outer segment of photoreceptors and RPE cells are coordinated with blood supply through the CC, and such a coordinated activity largely depends on the synchronized autophagy status between all these cell types. In fact, (1) light synchronizes autophagy by epigenetic regulation of 23 genes, all coding for autophagy proteins. As a consequence, more autophagy structures are generated during periods of light within the “inner choroid/outer retina” neurovascular unit, according to the “*momenta*” of light exposure. This is in line with the fact that autophagy in the retina is induced during the process of photo-transduction. These autophagy-related, light-induced proteins include “ezrin” inhibition (2), which removes lysosomal inhibition (3), thus making the autophagy pathway faster. Remarkably, the protein RPEDF is also induced (4), which is key in regulating the process of vision. This produces a physiological blood flow which inhibits abnormal angiogenesis (5) and increases blood supply (6), which occurs concomitantly with light exposure and autophagy induction.

This concept of neurovascular unit should be considered as an orchestrated activity of the blood vessels in the inner choroid to supply the metabolic demand of the RPE cells and outer photoreceptors in a way which is coordinated. In this way, when a high energy demand in the outer retina occurs, a cross-talk between RPE and inner choroid generates an appropriate modulation of blood flow and clearance of retina-dependent catabolites. Similarly, the status of the inner choroid is expected to modulate the activation of the outer retina through a specific signaling. In this way, the inner choroid/outer retina unit should be considered as a functional unit, meaning an area where the RPE and the inner choroid work in synergy. This hypothesis makes the inner choroid/outer retina unit similar to the neurovascular unit recently described in the CNS [113]. In line with this, in the retina the rate of autophagy within rods varies according to the light cycle being diurnally regulated [114]. This occurs according to the metabolic requests induced by vision. In this way, it is possible to define a so-called “inner choroid/outer retina” neurovascular unit, where the activity of the outer segment of the photoreceptors and RPE cells are coordinated with blood supply through the CC (Figure 7).

This in turn depends on the activity of pericytes and endothelial cells. The kernel of such a coordinated activity may largely depend on a synchronized autophagy status between all these cell types.

### 7.2. The Stimulating Effects of Light on Autophagy

Light-induced autophagy activity may be the key in triggering such a coordination. This may explain why autophagy machinery in the retina is finely tuned at the level of gene and protein expression to allow visual acuity [55,115]. In fact, more autophagy structures are generated within the “inner choroid/outer retina” neurovascular unit during periods of light. Such a cycle does not occur according to a circadian rhythm, but is directly stimulated by the “momenta” of light exposure [114]. This is confirmed by the fact that, when measuring autophagy in the retina, this is induced during the process of photo-transduction. In fact, autophagy stimulation in the outer retina generates a protein named RPEDF, which is key in regulating the process of vision [115].

The hypothesis of a cyclic regulation of the “inner choroid/outer retina” neurovascular unit by the autophagy status is further confirmed by the studies of Datta and colleagues [116], who attributed fluctuations in autophagy and blood supply to specific variations of metabolic demands in the outer retina. This is expected when considering the need of active autophagy to phagocytose the outer segment of photoreceptors and the cyclic metabolism of all-trans retinal by RPE cells following photo-transduction. The role of light in inducing the timing and spacing of autophagy activation applies to long wave-lengths. However, this also extends to the deleterious effects of short wave-length light to counteract the cyclic production of photo-induced oxidative stress.

Thus, it is not surprising that daily light, as much as artificial light, depending on their specific wave-lengths, may have robust epigenetic effects in the outer retina. This is needed to cope with light-induced metabolic demand, the process of vision, and oxidative damage. In fact, light regulates the expression of 23 autophagy-related genes [117]. In detrimental conditions, following short-wave length light or in the context of systemic metabolic disorders when autophagy is inhibited, light-induced epigenetic cycling of autophagy is attenuated.

The specific role of autophagy in the visual process also relies on the downstream between autophagosomes with lysosomes. In fact, Santo and Conte [63] indicate how critical is the final lysosomal step in providing an effective metabolic support for photoreceptors to respond to light exposure. We expect (and dedicated experiments are needed) that both timing and spacing of cyclic regulation of autophagy may occur in alternating retinal fields, likely to match the ON/OFF mosaic of retinal receptive fields. Accordingly, this may depend on the activation of either excitatory or inhibitory receptors to glutamate, whose release is inhibited by the effects of light. In this scenario, an ON retinal field should undergo downstream autophagy activation when impacted by light, whereas an OFF retinal field should feature autophagy suppression following the same light exposure. As shown by Intartaglia et al. [115], timing and spacing in retinal regulation of autophagy is under the influence of a specific molecule known as “ezrin”. Intartaglia et al. [115] consider the alternation of the autophagy status and light exposure as an appropriate stimulus to maintain retinal integrity. It is likely that pulses of light, owing to the right wavelength, may produce ezrin inhibition and autophagy activation to sustain a burst of trophic retinal activity. In fact, ezrin acting as an autophagy inhibitor prompts the external retina into a resting state and needs to be inhibited in order to stimulate autophagy and protect from neuronal damage. In fact, autophagy stimulation in the outer retina, within RPE, stimulates the expression of RPEDF, which restores the visual cycle [80,115].

The energy loss which accompanies autophagy activation and visual processing, combined with the need for light in order to activate autophagy in the ON retinal field, may explain why light exposure in order to be beneficial needs to be administered according to a pulsatile pattern, where blood flow fueling energy is coordinated. Similarly, due to the specific sensitivity of various photoreceptors to various wave-lengths, it is expected that, in order to sustain retinal integrity and visual functions, various wave-lengths should be combined during pulsatile light exposure, as recently suggested [17]. The marked autophagy demand in the outer retina is explained by the site-specificity of visual transduction and the recycling of visual structures, which is matched by strong mitochondrial activity and metabolic networks dedicated to neutralizing oxidative species. The coupling of these demands with appropriate functional changes in the CC is summarized by the novel concept of “inner choroid/outer retina neurovascular unit”, where alternating patches of the retina in alternating time frames are either inhibited or activated.

### 7.3. The Other Side of the Coin Provided by Light Stimulation

When discussing the effects of light in stimulating autophagy in the retina, it is important to emphasize which specific wave-lengths may sort these effects. In fact. the strong epigenetic stimulation of autophagy is induced in the range between amber, red and infra-red light [6,12,13,118,119,120,121] and the stimulation of retinal integrity mostly occurs following these wavelengths. Contrariwise, blue and cyanide light, mostly when delivered through LED, is detrimental for the structure of the retina and, rather, represents a major risk factor for the development of AMD [52,54,59,94,96,97,98,99,100,101,122,123]. In fact, these short wavelengths generate a massive amount of reactive oxygen species which engulf the autophagy machinery and produce mitochondrial damage, which in turn increase the amount of ROS. In addition, these wave-lengths sort an inhibitory effect on the autophagy pathway.

## 8. Downstream of Outer Retina

Although most studies focus on the transition zone between the epithelial/mesenchymal part of the eye where the choroid-retinal junction occurs [40,93,95], facts and hypotheses discussed so far are limited to the CC, the RPE and the external segment of the photoreceptor. Nonetheless, the relevance of autophagy downstream in the inner retina is reported [42]. This may rely on a number of biochemical issues. The role of autophagy within the whole photoreceptor cells is worth investigating. In fact, Intartaglia et al. [55,115] recently found that autophagy prevents cell death of retinal photoreceptors in the course of retinal degeneration.

In keeping with retinal neuroanatomy, a key role may be attributed to those glial cells which connect the outer retina with its inner part. In fact, the autophagy status within Muller cells is likely to be relevant to trans-synaptically modulating retinal homeostasis. It is shown that Muller cells, depending on their autophagy status, may induce neo-angiogenesis and inflammation, which occurs during retinal degeneration [124]. Even Muller cells’ viability is promoted by autophagy activation, which impedes Muller cells’ apoptosis during retinal degeneration [125].

The spreading of autophagy-dependent retinal activity to the inner retina may also occur as the consequence of the natural stream of excitation and inhibition through bipolar, amacrine, and down to ganglionic cells. In fact, the concept of retinal fields refers mostly to the downstream of glutamate receptors placed on bipolar cells, suggesting a patchy pattern of retinal degeneration, depending on this fact. It is not surprising, indeed, that retinal degeneration in the course of AMD is patchy by definition. The ability to establish a zonal distribution of light-induced autophagy modulation is expected to foster innovative research studies aimed at addressing site-specific autophagy failure in the course of retinal degeneration, in order to better comprehend the molecular mechanisms underlying site-specific retinal degenerative phenomena in AMD. This improved knowledge is expected to improve the treatment of retinal degeneration, mostly AMD, through a site-specific (macular) and wavelength-selective (red) light stimulation aimed at modifying the progression of the disease.

## Figures and Tables

**Table 1:** The amount of drusen does not necessarily correlate with visual symptoms.

	Patient 1	Patient 2
BCVA *	20/32	20/40
Drusenoid area	22.00 mm^2^	1.32 mm^2^
Central thickness	255 μm	216 μm
Metamorphopsia	0.4°	0.5°

* BCVA = Best corrected visual acuity.

## Data Availability

The data that supports the findings of this study are available from the corresponding author upon reasonable request.

## References

[1] Ramachandra Rao S., Fliesler S.J. (2022). Monitoring basal autophagy in the retina utilizing CAG-mRFP-EGFP-MAP1LC3B reporter mouse: Technical and biological considerations. Autophagy.

[2] Zhang Y., Cross S.D., Stanton J.B., Marmorstein A.D., Le Y.Z., Marmorstein L.Y. (2017). Early AMD-like defects in the RPE and retinal degeneration in aged mice with RPE-specific deletion of Atg5 or Atg7. Mol. Vis..

[3] Aoki H., Takada Y., Kondo S., Sawaya R., Aggarwal B.B., Kondo Y. (2007). Evidence that curcumin suppresses the growth of malignant gliomas in vitro and in vivo through induction of autophagy: Role of Akt and extracellular signal-regulated kinase signaling pathways. Mol. Pharmacol..

[4] Shinojima N., Yokoyama T., Kondo Y., Kondo S. (2007). Roles of the Akt/mTOR/p70S6K and ERK1/2 signaling pathways in curcumin-induced autophagy. Autophagy.

[5] Chang C.J., Lin J.F., Hsiao C.Y., Chang H.H., Li H.J., Chang H.H., Lee G.A., Hung C.F. (2017). Lutein Induces Autophagy via Beclin-1 Upregulation in IEC-6 Rat Intestinal Epithelial Cells. Am. J. Chin. Med..

[6] Comerota M.M., Tumurbaatar B., Krishnan B., Kayed R., Taglialatela G. (2019). Near Infrared Light Treatment Reduces Synaptic Levels of Toxic Tau Oligomers in Two Transgenic Mouse Models of Human Tauopathies. Mol. Neurobiol..

[7] Limanaqi F., Biagioni F., Busceti C.L., Ryskalin L., Polzella M., Frati A., Fornai F. (2019). Phytochemicals Bridging Autophagy Induction and Alpha-Synuclein Degradation in Parkinsonism. Int. J. Mol. Sci..

[8] Munia I., Gafray L., Bringer M.A., Goldschmidt P., Proukhnitzky L., Jacquemot N., Cercy C., Ramchani Ben Otman K., Errera M.H., Ranchon-Cole I. (2020). Cytoprotective Effects of Natural Highly Bio-Available Vegetable Derivatives on Human-Derived Retinal Cells. Nutrients.

[9] Ryskalin L., Biagioni F., Busceti C.L., Lazzeri G., Frati A., Fornai F. (2020). The Multi-Faceted Effect of Curcumin in Glioblastoma from Rescuing Cell Clearance to Autophagy-Independent Effects. Molecules.

[10] Pinelli R., Bertelli M., Scaffidi E., Bumah V.V., Biagioni F., Busceti C.L., Puglisi-Allegra S., Fornai F. (2021). The neurobiology of nutraceuticals combined with light exposure, a case report in the course of retinal degeneration. Arch. Ital. Biol..

[11] Ryskalin L., Puglisi-Allegra S., Lazzeri G., Biagioni F., Busceti C.L., Balestrini L., Fornasiero A., Leone S., Pompili E., Ferrucci M. (2021). Neuroprotective Effects of Curcumin in Methamphetamine-Induced Toxicity. Molecules.

[12] Stefenon L., Boasquevisque M., Garcez A.S., de Araújo V.C., Soares A.B., Santos-Silva A.R., Sperandio F., Brod J.M.M., Sperandio M. (2021). Autophagy upregulation may explain inhibition of oral carcinoma in situ by photobiomodulation in vitro. J. Photochem. Photobiol. B Biol..

[13] Yang K.L., Khoo B.Y., Ong M.T., Yoong I.C.K., Sreeramanan S. (2021). In vitro anti-breast cancer studies of LED red light therapy through autophagy. Breast Cancer.

[14] Algan A.H., Gungor-Ak A., Karatas A. (2022). Nanoscale Delivery Systems of Lutein: An Updated Review from a Pharmaceutical Perspective. Pharmaceutics.

[15] Dlamini M.B., Bao S., Gao Z., Mei J., Ge H., Jiang L., Geng C., Li Q., Shi X., Liu Y. (2022). Curcumin attenuates Cr (VI)-induced cell growth and migration by targeting autophagy-dependent reprogrammed metabolism. J. Biochem. Mol. Toxicol..

[16] Li X., Holt R.R., Keen C.L., Morse L.S., Zivkovic A.M., Yiu G., Hackman R.M. (2022). Potential roles of dietary zeaxanthin and lutein in macular health and function. Nutr. Rev..

[17] Pinelli R., Berti C., Scaffidi E., Lazzeri G., Bumah V.V., Ruffoli R., Biagioni F., Busceti C.L., Puglisi-Allegra S., Fornai F. (2022). Combined pulses of light and sound in the retina with nutraceuticals may enhance the recovery of foveal holes. Arch. Ital. Biol..

[18] Jin Q.H., Hu X.J., Zhao H.Y. (2022). Curcumin activates autophagy and attenuates high glucose-induced apoptosis in HUVECs through the ROS/NF-κB signaling pathway. Exp. Ther. Med..

[19] Hyttinen J.M.T., Błasiak J., Niittykoski M., Kinnunen K., Kauppinen A., Salminen A., Kaarniranta K. (2017). DNA damage response and autophagy in the degeneration of retinal pigment epithelial cells-Implications for age-related macular degeneration (AMD). Ageing Res. Rev..

[20] Jarrett S.G., Boulton M.E. (2012). Consequences of oxidative stress in age-related macular degeneration. Mol. Asp. Med..

[21] Kaarniranta K., Sinha D., Blasiak J., Kauppinen A., Veréb Z., Salminen A., Boulton M.E., Petrovski G. (2013). Autophagy and heterophagy dysregulation leads to retinal pigment epithelium dysfunction and development of age-related macular degeneration. Autophagy.

[22] Querques G., Rosenfeld P.J., Cavallero E., Borrelli E., Corvi F., Querques L., Bandello F.M., Zarbin M.A. (2014). Treatment of dry age-related macular degeneration. Ophthalmic Res..

[23] Bales K.L., Gross A.K. (2016). Aberrant protein trafficking in retinal degenerations: The initial phase of retinal remodeling. Exp. Eye Res..

[24] Arcella A., Biagioni F., Oliva A.M., Bucci D., Frati A., Esposito V., Cantore G., Giangaspero F., Fornai F. (2013). Rapamycin inhibits the growth of glioblastoma. Brain Res..

[25] Madeo F., Eisenberg T., Kroemer G. (2009). Autophagy for the avoidance of neurodegeneration. Genes Dev..

[26] Sethna S., Scott P.A., Giese A.P.J., Duncan T., Jian X., Riazuddin S., Randazzo P.A., Redmond T.M., Bernstein S.L., Riazuddin S. (2021). CIB2 regulates mTORC1 signaling and is essential for autophagy and visual function. Nat. Commun..

[27] Yefimova M.G. (2023). Myelinosome organelles in pathological retinas: Ubiquitous presence and dual role in ocular proteostasis maintenance. Neural Regen. Res..

[28] Ferrington D.A., Sinha D., Kaarniranta K. (2016). Defects in retinal pigment epithelial cell proteolysis and the pathology associated with age-related macular degeneration. Prog. Retin. Eye Res..

[29] Vessey K.A., Jobling A.I., Tran M.X., Wang A.Y., Greferath U., Fletcher E.L. (2022). Treatments targeting autophagy ameliorate the age-related macular degeneration phenotype in mice lacking APOE (apolipoprotein E). Autophagy.

[30] Nag T.C., Gorla S., Kumari C., Roy T.S. (2021). Aging of the human choriocapillaris: Evidence that early pericyte damage can trigger endothelial changes. Exp. Eye Res..

[31] Lengyel I., Tufail A., Hosaini H.A., Luthert P., Bird A.C., Jeffery G. (2004). Association of drusen deposition with choroidal intercapillary pillars in the aging human eye. Investig. Opthalmol. Vis. Sci..

[32] Mullins R.F., Johnson M.N., Faidley E.A., Skeie J.M., Huang J. (2011). Choriocapillaris vascular dropout related to density of drusen in human eyes with early age-related macular degeneration. Investig. Opthalmol. Vis. Sci..

[33] Terman A., Brunk U.T. (2004). Lipofuscin. Int. J. Biochem. Cell Biol..

[34] Kaemmerer E., Schutt F., Krohne T.U., Holz F.G., Kopitz J. (2007). Effects of lipid peroxidation-related protein modifications on RPE lysosomal functions and POS phagocytosis. Investig. Opthalmol. Vis. Sci..

[35] Kaarniranta K., Salminen A., Eskelinen E.-L., Kopitz J. (2009). Heat-shock proteins as gatekeepers, of proteolytic pathways: Implications for age-related macular degeneration. Ageing Res. Rev..

[36] Zhang M.Y., Zhu L., Bao X., Xie T.H., Cai J., Zou J., Wang W., Gu S., Li Y., Li H.Y. (2022). Inhibition of Drp1 ameliorates diabetic retinopathy by regulating mitochondrial homeostasis. Exp. Eye Res..

[37] Edwards M., Lutty G.A. (2021). Bruch’s Membrane and the Choroid in Age-Related Macular Degeneration. Adv. Exp. Med. Biol..

[38] Cai J., Zhang H., Zhang Y.F., Zhou Z., Wu S. (2019). MicroRNA-29 enhances autophagy and cleanses exogenous mutant αB-crystallin in retinal pigment epithelial cells. Exp. Cell Res..

[39] Liu J., Copland D.A., Theodoropoulou S., Chiu H.A., Barba M.D., Mak K.W., Mack M., Nicholson L.B., Dick A.D. (2016). Impairing autophagy in retinal pigment epithelium leads to inflammasome activation and enhanced macrophage-mediated angiogenesis. Sci. Rep..

[40] Pinelli R., Biagioni F., Limanaqi F., Bertelli M., Scaffidi E., Polzella M., Busceti C.L., Fornai F. (2020). A Re-Appraisal of Pathogenic Mechanisms Bridging Wet and Dry Age-Related Macular Degeneration Leads to Reconsider a Role for Phytochemicals. Int. J. Mol. Sci..

[41] Du J.H., Li X., Li R., Cheng B.X., Kuerbanjiang M., Ma L. (2017). Role of Autophagy in Angiogenesis Induced by a High-Glucose Condition in RF/6A Cells. Ophthalmologica.

[42] Pinelli R., Bertelli M., Scaffidi E., Busceti C.L., Biagioni F., Fornai F. (2021). Exosomes and alpha-synuclein within retina from autophagy to protein spreading in neurodegeneration. Arch. Ital. Biol..

[43] Hyttinen J.M.T., Niittykoski M., Salminen A., Kaarniranta K. (2013). Maturation of autophagosomes and endosomes: A key role for Rab7. Biochim. Biophys. Acta (BBA)-Mol. Cell Res..

[44] Hyttinen J.M.T., Amadio M., Viiri J., Pascale A., Salminen A., Kaarniranta K. (2014). Clearance of misfolded and aggregated proteins by aggrephagy and implication for aggregation diseases. Ageing Res. Rev..

[45] Kaarniranta K., Tokarz P., Koskela A., Paterno J., Blasiak J. (2017). Autophagy regulates death of retinal pigment epithelium cells in age-related macular degeneration. Cell Biol. Toxicol..

[46] Luzio J.P., Pryor P.R., Bright N.A. (2007). Lysosomes: Fusion and function. Nat. Rev. Mol. Cell Biol..

[47] Ishibashi T., Murata T., Hangai M., Nagai R., Horiuchi S., Lopez P.F., Hinton D.R., Ryan S.J. (1998). Advanced glycation end products in age-related macular degeneration. Arch. Ophthalmol..

[48] Lee E.J., Kim J.Y., Oh S.H. (2016). Advanced glycation end products (AGEs) promote melanogenesis through receptor for AGEs. Sci. Rep..

[49] Fang J., Ouyang M., Qu Y., Wang M., Huang X., Lan J., Lai W., Xu Q. (2022). Advanced Glycation End Products Promote Melanogenesis by Activating NLRP3 Inflammasome in Human Dermal Fibroblasts. J. Investig. Dermatol..

[50] Spraul C.W., Lang G.E., Grossniklaus H.E., Lang G.K. (1999). Histologic and morphometric analysis of the choroid, Bruch’s membrane, and retinal pigment epithelium in postmortem eyes with age-related macular degeneration and histologic examination of surgically excised choroidal neovascular membranes. Surv. Ophthalmol..

[51] Jager R.D., Mieler W.F., Miller J.W. (2008). Age-related macular degeneration. N. Engl. J. Med..

[52] Lin C.W., Yang C.M., Yang C.H. (2020). Protective Effect of Astaxanthin on Blue Light Light-Emitting Diode-Induced Retinal Cell Damage via Free Radical Scavenging and Activation of PI3K/Akt/Nrf2 Pathway in 661W Cell Model. Mar. Drugs..

[53] Nita M., Grzybowski A. (2020). Interplay between reactive oxygen species and autophagy in the course of age-related macular degeneration. EXCLI J..

[54] Cheng K.C., Hsu Y.T., Liu W., Huang H.L., Chen L.Y., He C.X., Sheu S.J., Chen K.J., Lee P.Y., Lin Y.H. (2021). The Role of Oxidative Stress and Autophagy in Blue-Light-Induced Damage to the Retinal Pigment Epithelium in Zebrafish In Vitro and In Vivo. Int. J. Mol. Sci..

[55] Intartaglia D., Giamundo G., Conte I. (2022). Autophagy in the retinal pigment epithelium: A new vision and future challenges. FEBS J..

[56] Pinelli R., Biagioni F., Scaffidi E., Vakunseth Bumah V., Busceti C.L., Puglisi-Allegra S., Lazzeri G., Fornai F. (2022). The potential effects of nutrients and light on autophagy-mediated visual function and clearance of retinal aggregates. Arch. Ital. Biol..

[57] Chan C.M., Huang D.Y., Sekar P., Hsu S.H., Lin W.W. (2019). Reactive oxygen species-dependent mitochondrial dynamics and autophagy confer protective effects in retinal pigment epithelial cells against sodium iodate-induced cell death. J. Biomed. Sci..

[58] Kim J.Y., Park S., Park H.J., Kim S.H., Lew H., Kim G.J. (2021). PEDF-Mediated Mitophagy Triggers the Visual Cycle by Enhancing Mitochondrial Functions in a H_2_O_2_-Injured Rat Model. Cells.

[59] Wang L., Yu X., Zhang D., Wen Y., Zhang L., Xia Y., Chen J., Xie C., Zhu H., Tong J. (2023). b Long-term blue light exposure impairs mitochondrial dynamics in the retina in light-induced retinal degeneration in vivo and in vitro. J. Photochem. Photobiol. B Biol..

[60] Zou G.P., Wang T., Xiao J.X., Wang X.Y., Jiang L.P., Tou F.F., Chen Z.P., Qu X.H., Han X.J. (2023). Lactate protects against oxidative stress-induced retinal degeneration by activating autophagy. Free. Radic. Biol. Med..

[61] Notomi S., Ishihara K., Efstathiou N.E., Lee J.J., Hisatomi T., Tachibana T., Konstantinou E.K., Ueta T., Murakami Y., Maidana D.E. (2019). Genetic LAMP2 deficiency accelerates the age-associated formation of basal laminar deposits in the retina. Proc. Natl. Acad. Sci. USA.

[62] Lee J.J., Ishihara K., Notomi S., Efstathiou N.E., Ueta T., Maidana D., Chen X., Iesato Y., Caligiana A., Vavvas D.G. (2020). Lysosome-associated membrane protein-2 deficiency increases the risk of reactive oxygen species-induced ferroptosis in retinal pigment epithelial cells. Biochem. Biophys. Res. Commun..

[63] Santo M., Conte I. (2021). Emerging Lysosomal Functions for Photoreceptor Cell Homeostasis and Survival. Cells.

[64] Matsumoto B., Defoe D.M., Besharse J.C. (1987). Membrane turnover in rod photoreceptors: Ensheathment and phagocytosis of outer segment distal tips by pseudopodia of the retinal pigment epithelium. Proc. R. Soc. London. Ser. B Boil. Sci..

[65] Kim J.Y., Zhao H., Martinez J., Doggett T.A., Kolesnikov A.V., Tang P.H., Ablonczy Z., Chan C.C., Zhou Z., Green D.R. (2013). Noncanonical autophagy promotes the visual cycle. Cell.

[66] Kwon W., Freeman S.A. (2020). Phagocytosis by the Retinal Pigment Epithelium: Recognition, Resolution, Recycling. Front. Immunol..

[67] Ferguson T.A., Green D.R. (2014). Autophagy and phagocytosis converge for better vision. Autophagy.

[68] Frost L.S., Lopes V.S., Bragin A., Reyes-Reveles J., Brancato J., Cohen A., Mitchell C.H., Williams D.S., Boesze-Battaglia K. (2015). The Contribution of Melanoregulin to Microtubule-Associated Protein 1 Light Chain 3 (LC3) Associated Phagocytosis in Retinal Pigment Epithelium. Mol. Neurobiol..

[69] Muniz-Feliciano L., Doggett T.A., Zhou Z., Ferguson T.A. (2017). RUBCN/rubicon and EGFR regulate lysosomal degradative processes in the retinal pigment epithelium (RPE) of the eye. Autophagy.

[70] Mitter S.K., Song C., Qi X., Mao H., Rao H., Akin D., Lewin A., Grant M., Dunn W., Ding J. (2014). Dysregulated autophagy in the RPE is associated with increased susceptibility to oxidative stress and AMD. Autophagy.

[71] Boyer N.P., Tang P.H., Higbee D., Ablonczy Z., Crouch R.K., Koutalos Y. (2012). Lipofuscin and A2E accumulate with age in the retinal pigment epithelium of Nrl-/- mice. Photochem. Photobiol..

[72] Kaarniranta K., Hyttinen J., Ryhänen T., Viiri J., Paimela T., Toropainen E., Sorri I., Salminen A. (2010). Mechanisms of protein aggregation in the retinal pigment epithelial cells. Front. Biosci..

[73] Blazer A., Qian Y., Schlegel M.P., Algasas H., Buyon J.P., Cadwell K., Cammer M., Heffron S.P., Liang F.X., Mehta-Lee S. (2022). APOL_1_ variant-expressing endothelial cells exhibit autophagic dysfunction and mitochondrial stress. Front. Genet..

[74] Lenzi P., Marongiu R., Falleni A., Gelmetti V., Busceti C.L., Michiorri S., Valente E.M., Fornai F. (2012). A subcellular analysis of genetic modulation of PINK1 on mitochondrial alterations, autophagy and cell death. Arch. Ital. Biol..

[75] Liegl R., Koenig S., Siedlecki J., Haritoglou C., Kampik A., Kernt M. (2014). Temsirolimus inhibits proliferation and migration in retinal pigment epithelial and endothelial cells via mTOR inhibition and decreases VEGF and PDGF expression. PLoS ONE.

[76] Sghaier R., Perus M., Cornebise C., Courtaut F., Scagliarini A., Olmiere C., Aires V., Hermetet F., Delmas D. (2022). Resvega, a Nutraceutical Preparation, Affects NFκB Pathway and Prolongs the Anti-VEGF Effect of Bevacizumab in Undifferentiated ARPE-19 Retina Cells. Int. J. Mol. Sci..

[77] Wang N., Zhang C., Xu Y., Tan H.Y., Chen H., Feng Y. (2021). Berberine improves insulin-induced diabetic retinopathy through exclusively suppressing Akt/mTOR-mediated HIF-1α/VEGF activation in retina endothelial cells. Int. J. Biol. Sci..

[78] Asani B., Siedlecki J., Wertheimer C., Liegl R., Wolf A., Ohlmann A., Priglinger S., Priglinger C. (2022). Anti-angiogenic properties of rapamycin on human retinal pericytes in an in vitro model of neovascular AMD via inhibition of the mTOR pathway. BMC Ophthalmol..

[79] Xia W., Li C., Chen Q., Huang J., Zhao Z., Liu P., Xu K., Li L., Hu F., Zhang S. (2022). Intravenous route to choroidal neovascularization by macrophage-disguised nanocarriers for mTOR modulation. Acta Pharm. Sin. B.

[80] Tian R., Deng A., Pang X., Chen Y., Gao Y., Liu H., Hu Z. (2022). VR-10 polypeptide interacts with CD36 to induce cell apoptosis and autophagy in choroid-retinal endothelial cells: Identification of VR-10 as putative novel therapeutic agent for choroid neovascularization (CNV) treatment. Peptides.

[81] Xie L., Ji X., Tu Y., Wang K., Zhu L., Zeng X., Wang X., Zhang J., Zhu M. (2020). MLN4924 inhibits hedgehog signaling pathway and activates autophagy to alleviate mouse laser-induced choroidal neovascularization lesion. Biomed. Pharmacother..

[82] Curcio C.A., Millican C.L. (1999). Basal linear deposit and large drusen are specific for early age-related maculopathy. Arch. Ophthalmol..

[83] Choi E.Y., Kim H.R., Jung J., Byeon S.H., Kim S.S., Kim M. (2023). Bilateral Macular Choroidal Abnormalities with Drusenoid Deposits in Patients with Unilateral Peripheral Exudative Hemorrhagic Chorio-retinopathy. Retina.

[84] Ma J., Teng Y., Huang Y., Tao X., Fan Y. (2022). Autophagy plays an essential role in ultraviolet radiation-driven skin photoaging. Front. Pharmacol..

[85] Li B., Zhang Z., Wang H., Zhang D., Han T., Chen H., Chen J., Chen Z., Xie Y., Wang L. (2022). Melatonin promotes peripheral nerve repair through Parkin-mediated mitophagy. Free. Radic. Biol. Med..

[86] Ning R., Li Y., Du Z., Li T., Sun Q., Lin L., Xu Q., Duan J., Sun Z. (2021). The mitochondria-targeted antioxidant MitoQ attenuated PM_2.5_-induced vascular fibrosis via regulating mitophagy. Redox Biol..

[87] Gross-Jendroska M., Lui G.M., Song M.K., Stern R. (1992). Retinal pigment epithelium-stromal interactions modulate hyaluronic acid deposition. Investig. Opthalmol. Vis. Sci..

[88] Congdon N., O’Colmain B., Klaver C.C., Klein R., Muñoz B., Friedman D.S., Kempen J., Taylor H.R., Mitchell P. (2004). Causes and prevalence of visual impairment among adults in the United States. Arch. Ophthalmol..

[89] Pascolini D., Mariotti S.P., Pokharel G.P., Pararajasegaram R., Etya’ale D., Négrel A.D., Resniko S. (2004). 2002 global update of available data on visual impairment: A compilation of population-based prevalence studies. Ophthalmic Epidemiol..

[90] De Jong P.T. (2006). Age-related macular degeneration. N. Engl. J. Med..

[91] Wong W.L., Su X., Li X., Cheung C.M., Klein R., Cheng C.Y., Wong T.Y. (2014). Global prevalence of age-related macular degeneration and disease burden projection for 2020 and 2040: Asystematic review and meta-analysis. Lancet Glob. Health.

[92] Wong T.Y., Chakravarthy U., Klein R., Mitchell P., Zlateva G., Buggage R., Fahrbach K., Probst C., Sledge I. (2008). The natural history and prognosis of neovascular age-related macular degeneration: A systematic review of the literature and meta-analysis. Ophthalmology.

[93] Pinelli R., Bertelli M., Scaffidi E., Fulceri F., Busceti C.L., Biagioni F., Fornai F. (2020). Measurement of drusen and their correlation with visual symptoms in patients affected by age-related macular degeneration. Arch. Ital. Biol..

[94] Tisi A., Flati V., Delle Monache S., Lozzi L., Passacantando M., Maccarone R. (2020). Nanoceria Particles Are an Eligible Candidate to Prevent Age-Related Macular Degeneration by Inhibiting Retinal Pigment Epithelium Cell Death and Autophagy Alterations. Cells.

[95] Kaarniranta K., Blasiak J., Liton P., Boulton M., Klionsky D.J., Sinha D. (2023). Autophagy in age-related macular degeneration. Autophagy.

[96] Jaadane I., Villalpando Rodriguez G.E., Boulenguez P., Chahory S., Carré S., Savoldelli M., Jonet L., Behar-Cohen F., Martinsons C., Torriglia A. (2017). Effects of white light-emitting diode (LED) exposure on retinal pigment epithelium in vivo. J. Cell Mol. Med..

[97] Hall H., Ma J., Shekhar S., Leon-Salas W.D., Weake V.M. (2018). Blue light induces a neuroprotective gene expression program in Drosophila photoreceptors. BMC Neurosci..

[98] Tao J.X., Zhou W.C., Zhu X.G. (2019). Mitochondria as Potential Targets and Initiators of the Blue Light Hazard to the Retina. Oxidative Med. Cell. Longev..

[99] International Commission on Non-Ionizing Radiation Protection (ICNIRP) (2020). Light-Emitting Diodes (LEDS): Implications for Safety. Health Phys..

[100] Otsu W., Ishida K., Nakamura S., Shimazawa M., Tsusaki H., Hara H. (2020). Blue light-emitting diode irradiation promotes transcription factor EB-mediated lysosome biogenesis and lysosomal cell death in murine photoreceptor-derived cells. Biochem. Biophys. Res. Commun..

[101] Lin Y.H., Sheu S.J., Liu W., Hsu Y.T., He C.X., Wu C.Y., Chen K.J., Lee P.Y., Chiu C.C., Cheng K.C. (2023). Retinal protective effect of curcumin metabolite hexahydrocurcumin against blue light-induced RPE damage. Phytomedicine.

[102] Kang Q., Dai H., Jiang S., Yu L. (2022). Advanced glycation end products in diabetic retinopathy and phytochemical therapy. Front. Nutr..

[103] Takkar B., Sheemar A., Jayasudha R., Soni D., Narayanan R., Venkatesh P., Shivaji S., Das T. (2022). Unconventional avenues to decelerate diabetic retinopathy. Surv. Ophthalmol..

[104] Lin J.B., Halawa O.A., Husain D., Miller J.W., Vavvas D.G. (2022). Dyslipidemia in age-related macular degeneration. Eye.

[105] Pinheiro R.L., Marques J.P., Murta J.N. (2023). “Lipoid” Macular Edema in Familial Hypertriglyceridemia and Retinal Dystrophy. Ophthalmol. Retin..

[106] Campos M.M., Abu-Asab M.S. (2017). Loss of endothelial planar cell polarity and cellular mclearance mechanisms in age-related macular degeneration. Ultrastruct. Pathol..

[107] Torisu K., Singh K.K., Torisu T., Lovren F., Liu J., Pan Y., Quan A., Ramadan A., Al-Omran M., Pankova N. (2016). Intact endothelial autophagy is required to maintain vascular lipid homeostasis. Aging Cell.

[108] Chang N.C. (2020). Autophagy and Stem Cells: Self-Eating for Self-Renewal. Front. Cell Dev. Biol..

[109] Zhang M., Chu Y., Mowery J., Konkel B., Galli S., Theos A.C., Golestaneh N. (2018). Pgc-1α repression and high-fat diet induce age-related macular degeneration-like phenotypes in mice. Dis. Model. Mech..

[110] Kauppinen A., Paterno J.J., Blasiak J., Salminen A., Kaarniranta K. (2016). Inflammation and its role in age-related macular degeneration. Cell Mol. Life Sci..

[111] Golestaneh N., Chu Y., Xiao Y.Y., Stoleru G.L., Theos A.C. (2017). Dysfunctional autophagy in RPE, a contributing factor in age-related macular degeneration. Cell Death Dis..

[112] Wang A.L., Lukas T.J., Yuan M., Du N., Tso M.O., Neufeld A.H. (2009). Autophagy and exosomes in the aged retinal pigment epithelium: Possible relevance to drusen formation and age-related macular degeneration. PLoS ONE.

[113] Schaeffer S., Iadecola C. (2021). Revisiting the neurovascular unit. Nat. Neurosci..

[114] Wen R.H., Stanar P., Tam B., Moritz O.L. (2019). Autophagy in Xenopus laevis rod photoreceptors is independently regulated by phototransduction and misfolded RHO ^P23H^. Autophagy.

[115] Intartaglia D., Giamundo G., Naso F., Nusco E., Di Giulio S., Salierno F.G., Polishchuk E., Conte I. (2022). Induction of Autophagy Promotes Clearance of RHO^P23H^ Aggregates and Protects from Retinal Degeneration. Front. Aging Neurosci..

[116] Datta S., Cano M., Satyanarayana G., Liu T., Wang L., Wang J., Cheng J., Itoh K., Sharma A., Bhutto I. (2022). Mitophagy initiates retrograde mitochondrial-nuclear signaling to guide retinal pigment cell heterogeneity. Autophagy.

[117] Wang N., Wei L., Liu D., Zhang Q., Xia X., Ding L., Xiong S. (2022). Identification and Validation of Autophagy-Related Genes in Diabetic Retinopathy. Front. Endocrinol..

[118] Rojas J.C., Lee J., John J.M., Gonzalez-Lima F. (2008). Neuroprotective effects of near infrared light in an in vivo model of mitochondrial optic neuropathy. J. Neurosci..

[119] Rojas J.C., Gonzalaz-Lima F. (2011). Low level light therapy of the eye and brain. Eye Brain..

[120] Tata D.B., Waynant R.W. (2011). Laser therapy: A review of its mechanism of action and potential medical applications. Laser Photonics Rev..

[121] Choi M.S., Kim H.J., Ham M., Choi D.H., Lee T.R., Shin D.W. (2016). Amber Light (590 nm) Induces the Breakdown of Lipid Droplets through Autophagy-Related Lysosomal Degradation in Differentiated Adipocytes. Sci. Rep..

[122] Ren C., Hu W., Wei Q., Cai W., Jin H., Yu D., Liu C., Shen T., Zhu M., Liang X. (2021). MicroRNA-27a Promotes Oxidative-Induced RPE Cell Death through Targeting FOXO1. Biomed. Res. Int..

[123] Abdouh M., Lu M., Chen Y., Goyeneche A., Burnier J.V., Burnier M.N. (2022). Filtering blue light mitigates the deleterious effects induced by the oxidative stress in human retinal pigment epithelial cells. Exp. Eye Res..

[124] Subirada P.V., Vaglienti M.V., Joray M.B., Paz M.C., Barcelona P.F., Sánchez M.C. (2022). Rapamycin and Resveratrol Modulate the Gliotic and Pro-Angiogenic Response in Müller Glial Cells Under Hypoxia. Front. Cell Dev. Biol..

[125] Wang L., Sun X., Zhu M., Du J., Xu J., Qin X., Xu X., Song E. (2019). Epigallocatechin-3-gallate stimulates autophagy and reduces apoptosis levels in retinal Müller cells under high-glucose conditions. Exp. Cell Res..

